# Epigallocatechin-3-gallate (EGCG) protects skin cells from ionizing radiation via heme oxygenase-1 (HO-1) overexpression

**DOI:** 10.1093/jrr/rru047

**Published:** 2014-06-26

**Authors:** Wei Zhu, Jing Xu, Yangyang Ge, Han Cao, Xin Ge, Judong Luo, Jiao Xue, Hongying Yang, Shuyu Zhang, Jianping Cao

**Affiliations:** 1 School of Radiation Medicine and Protection and Jiangsu Provincial Key Laboratory of Radiation Medicine and Protection, Soochow University, Suzhou 215123, China; 2 Collaborative Innovation Center of Radiation Medicine of Jiangsu Higher Education Institutions and School for Radiological and Interdisciplinary Sciences (RAD-X), Soochow University, No. 199 Ren'ai Road, Suzhou 215123, China; 3 Department of Radiotherapy, Changzhou Tumor Hospital, Soochow University, Changzhou, 213001, China

**Keywords:** epigallocatechin-3-gallate (EGCG), radiation-induced skin injury, skin HaCaT cells, heme oxygenase-1 (HO-1)

## Abstract

Epigallocatechin-3-gallate (EGCG), the major polyphenolic constituent of green tea, is a potent antioxidant and free radical scavenger that may have therapeutic applications for the treatment of many disorders. Radiation therapy is widely used for the treatment of various types of cancers; however, radiation-induced skin injury remains a serious concern. EGCG has not yet been reported as protecting skin cells against ionizing radiation. In the present study, we investigated whether EGCG confers cytoprotection against ionizing radiation. We found that, compared with the control, pretreatment with EGCG significantly enhanced the viability of human skin cells that were irradiated with X-rays, and decreased apoptosis induced by X-ray irradiation. Mito-Tracker assay showed that EGCG suppressed the damage to mitochondria induced by ionizing radiation via upregulation of SOD2. Reactive oxygen species (ROS) in HaCaT cells were significantly reduced when pretreated with EGCG before irradiation. Radiation-induced γH2AX foci, which are representative of DNA double-strand breaks, were decreased by pretreatment with EGCG. Furthermore, EGCG induced the expression of the cytoprotective molecule heme oxygenase-1 (HO-1) in a dose-dependent manner via transcriptional activation. HO-1 knockdown or treatment with the HO-1 inhibitor tin protoporphyrin (SnPPIX) reversed the protective role of EGCG, indicating an important role for HO-1. These results suggest that EGCG offers a new strategy for protecting skin against ionizing radiation.

## INTRODUCTION

Since ancient times, green tea (*Camellia sinensis*) has been considered a health-promoting beverage. Epigallocatechin-3-gallate (EGCG) is the most abundant catechin in green tea and is a traditional anti-oxidative free radical scavenger [1, 2]. EGCG has been demonstrated to have anti-inflammatory [3], anti-arthritic [4, 5], antibacterial [6], anti-angiogenic [7] anti-aging [8], antiviral [9] and neuroprotective effects [10] that may have therapeutic applications in the treatment of many disorders, including atherosclerosis [11] and cardiovascular and metabolic diseases [12, 13]. The major mechanism for the EGCG-mediated beneficial effects is thought to be related to its antioxidant structure. The phenolic groups of EGCG serve as electron donors that can give up an electron or a hydrogen atom, and the phenolic molecule is capable of making an internal adjustment to stabilize the unpaired electron that results from the loss [14]. EGCG has been reported as increasing the levels of several anti-oxidant enzymes related to oxidative stress, including superoxide dismutases (SODs), c-GST, glutamate cysteine ligase and heme oxygenase-1 (HO-1), both *in vitro* and *in vivo* [15]. Among these enzymes, HO-1 is considered to be a cytoprotective protein. HO-1 catalyzes the heme ring conversion into carbon monoxide, free iron and biliverdin. HO-1 is strongly induced by various stimuli, including heat shock, metals, cytokines and oxidative stress [16, 17]. Of the multiple different green tea constituents, EGCG is reported to be the most potent inducer of HO-1 expression in an NF-E2-related factor-2 (Nrf2)-dependent manner [18].

Radiation therapy is widely used for the treatment of various types of cancer [19]. However, along with the destruction of tumors, surrounding normal tissues may also be injured, including brain, lung, intestine and skin. Skin covers the largest area of the body and functions to protect the body from all types of noxious substances [20]. Because skin is usually the first site of entry for external radiation particles in radiation treatment, variable degrees of skin reactions can occur. Serious radiation-induced skin injuries can cause severe pain, deformation, secondary infection, ulceration, and even necrosis when intolerable doses are administered [21]. It is reported that ∼87% of patients receiving radiotherapy suffer from erythema and radiation-induced skin damage [21]. Ionizing radiation is known to induce production of reactive oxidative species (ROS) (due to radiolysis of water and direct ionization of target molecules), which are comprised of superoxide, peroxynitrite, hydroxyl radicals and hydrogen peroxide [22]. All of these changes could result in oxidative damage and cytotoxicity to critical cellular biomacromolecules, including nucleic acids, proteins, and lipids [23].

Due to the antioxidant effect of EGCG, we hypothesize that EGCG may protect skin cells against ionizing radiation.

## MATERIALS AND METHODS

### Reagents

EGCG was purchased from Sigma–Aldrich (St Louis, MO, USA) and dissolved in dimethylsulfoxide (DMSO, Solon, OH, USA). siRNA targeting HO-1 was purchased from Santa Cruz Biotechnology (Santa Cruz, CA, USA). Antibodies against HO-1, Bax, Bcl-2, SOD1, SOD2 and the internal standard β-actin (all from Santa Cruz Biotechnology) were used for western blotting analysis. Tin protoporphyrin (SnPPIX) was obtained from Sigma–Aldrich (St Louis, MO, USA). γH2AX (pS139) antibody was purchased from Epitomics (Burlingame, CA, USA).

### Cell culture and irradiation

The human epidermal keratinocyte cell line HaCaT was maintained in Dulbecco's modified Eagle's medium (DMEM) supplemented with 10% (v/v) heat-inactivated fetal bovine serum and 1% (v/v) penicillin–streptomycin at 37°C in a humidified atmosphere containing 5% CO_2_. Cells were irradiated using an X-ray linear accelerator (RADSOURCE, GA, USA) at a fixed dose rate of 1.15 Gy/min. 5-Gy X-ray irradiation was chosen because it causes appropriate DNA damage [24, 25].

### MTT assay

The effect of EGCG on cell viability with radiation was measured by the MTT colorimetric assay. The concentration of DMSO in the medium was < 0.5% for all conditions. The MTT assay was carried out in 96-well plates. The HaCaT cells (4 × 10^5^ cells/well) were pretreated with various concentrations of EGCG 1 h prior to irradiation. Then, the medium was replaced by fresh medium and the cells were incubated for additional 24 or 48 h after radiation. Cells were incubated with 20 µl of 0.5 mg/ml MTT for 4 h. The supernatant was removed and 150 μl of DMSO was added to each well to dissolve the formazan for 10 min by vibration. The optical density (OD) value was measured using a microplate reader at the wavelength of 570 nm.

### Clonogenic assay

For standard clonogenic assays, stable cell lines were seeded into six-well plates at 200–2000 cells/well, depending on the dose of radiation. Cells were pretreated with 50 µM EGCG. The concentration of DMSO in the medium was < 0.5% for all conditions. Cells were irradiated using an X-ray linear accelerator (RADSOURCE, San Francisco, CA, USA) at a fixed dose rate of 1.15 Gy/min. After radiation, the drug-containing medium was immediately replaced by fresh medium. The cells were then grown for 7–10 d to allow for colony formation and were subsequently stained using crystal violet. Colonies consisting of 50 or more cells were counted as a clone.

### Immunofluorescence assay

HaCaT cells were plated on coverslips in six-well plates for 12 h, and then cells were pre-incubated with 50 µM EGCG 1 h before radiation exposure. The concentration of DMSO in the medium was < 0.5% for all conditions. The cells were washed with PBS, fixed using freshly prepared 4% paraformaldehyde and permeabilized with 0.5% Triton X-100 (Sigma, St Louis, MO, USA) after treatment. The γH2AX antibody was diluted 1:500 in 1% bovine serum albumin (BSA) in PBS and incubated for 12 h at 4°C, followed by incubation with the secondary antibody, fluorescein TRITC-conjugated goat anti-rabbit IgG (Southern Biotech, AL, USA), for 45 min at 37°C. The cells were counterstained using DAPI to visualize the cell nucleus and were observed using a fluorescence microscope (Leica SP2, Heidelberg, Germany).

### Reactive oxygen species generation assay

ROS levels of HaCaT were determined using the ROS-sensitive dye 2′,7′-dichlorofluorescein diacetate (DCF-DA), which is converted by ROS into the highly fluorescent 2′,7′-dichlorofluorescein (DCF). Cells were pretreated with EGCG (50 µM) for 1 h before irradiation. The concentration of DMSO in the medium was < 0.5% for all conditions. HaCaT cells were then washed with phosphate buffer (pH 7.4) and incubated with DCF-DA (10 μM) for 30 min. The level of DCF fluorescence, reflecting the concentration of ROS, was measured using a fluorescence microscope (Leica SP2, Heidelberg, Germany). Relative fluorescence intensity was calculated using ImageJ image analysis software (Bethesda, MD, USA).

### Cell apoptosis assay

We used two methods to detect cell apoptosis: DNA fragmentation and Annexin V-propidium iodide (PI) double-staining analysis. EGCG was added to cells 1 h before irradiation. The concentration of DMSO in the medium was < 0.5% for all conditions. The cells were further incubated for 48 h, followed by DNA extraction. The genomic DNA extraction kit was purchased from TianGen (Beijing, China). Isolated DNA, which was mainly derived from the apoptotic bodies that occurred in cells, was subjected to 1.2% agarose electrophoresis at 35 V for 4h. HaCaT cells were seeded at 1 × 10^5^/well in six-well plates. For Annexin V/PI double-staining analysis, cells were pretreated with EGCG 1 h before 20-Gy irradiation. Cells pretreated with DMSO served as negative controls. After 48 h, cells were stained with fluorescein FITC-conjugated Annexin V and PI (KeyGen, Nanjing, China). The analysis was performed using flow cytometry (Beckman-Coulter, Brea, CA, USA).

### Western blot

Lysates from HaCaT cells were prepared by incubating cells in lysis buffer (125 mM Tris–HCl, pH 6.8, 2% SDS and 10% v/v glycerol) at 4°C for 30 min. Protein concentration was determined by BCA protein assay (Bio-Rad, CA, USA). Then, 40 μg of cell lysate was subjected to 12% sodium dodecyl sulfate-polyacrylamide gel electrophoresis (SDS-PAGE). Following SDS-PAGE, the proteins were transferred onto a PVDF membrane (Millipore, Bedford, MA, USA), which was blocked with 10% dry milk in tris buffered saline with tween-20. The membrane was then probed with the indicated antibodies at 4°C for 12 h, followed by HRP-conjugated anti-rabbit or mouse IgG secondary antibody (Santa Cruz, CA, USA). The blots were incubated with detection reagent (ECL Advance Western Blotting Detection Kit, Amersham, Chicago, IL, USA), followed by exposure to a Hyperfilm ECL film (Pierce, Rockford, IL, USA).

### Mitochondria mass assessment

Mito-Tracker was used to determine the mitochondria mass of HaCaT cells. HaCaT cells were plated in 35-mm glass dishes. Cells were pretreated with EGCG for 1 h before 20-Gy X-ray irradiation. After treatment, cells were incubated for 30 min in the dark with Mito-Tracker red fluorescent stain (Molecular Probes, Eugene, OR, USA), which was dissolved in serum-free medium at 37°C. Hoechst stain was used to mark the nucleus. Relative fluorescence intensity was calculated with ImageJ image analysis software (Bethesda, MD, USA).

### Cell transfection and luciferase assays

For transfection, cells were transfected by Lipofectamine 2000 (Invitrogen, Carlsbad, CA) with plasmids or siRNAs. A luciferase reporter containing a fragment of the *HO-1* promoter (−1724 to +16, relative to the transcription start site) was constructed by Shanghai Biobuy Co. Ltd (Shanghai, China). For the luciferase assay, 1 µg of the reporter vector with the *HO-1* promoter was co-transfected with 50 ng of pRL-TK (Promega, Madison, WI, USA) to correct for transfection efficiency. Luciferase activity was measured with the Dual-Luciferase Reporter Assay System (Promega). Promoter activities were expressed as the ratio of *Firefly* luciferase to *Renilla* luciferase activities.

### Statistical analysis

Data were expressed as the mean ± standard error of the mean (SEM) of at least three independent experiments. Statistical analysis was performed using Student's *t*-test when only two groups were present or a one-way analysis of variance followed by the Student–Newman–Keuls test when more than two groups were compared. The radiation sensitivity enhancement ratio (SER) was measured according to the multitarget single hit model. Statistical analyses were conducted with SPSS 16.0 Software (SPSS, WA, USA). *P* < 0.05 was considered significant.

## RESULTS

### Increased viability of HaCaT cells by EGCG after irradiation

**Fig. 1.**
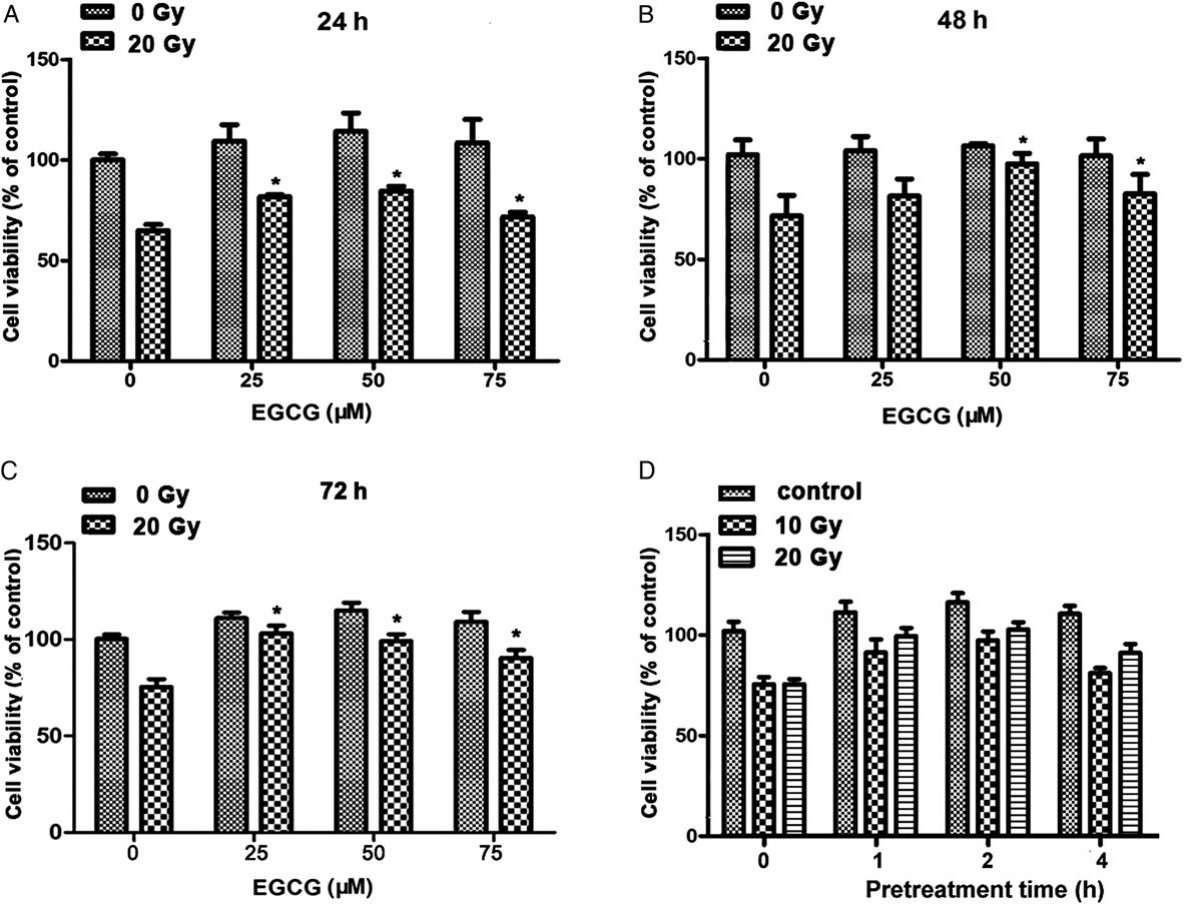
EGCG inhibits radiation-induced cytotoxicity in HaCaT cells. Cell viability was determined using the MTT assay. HaCaT cells were pretreated with the indicated concentrations of EGCG 1 h before irradiation. Then, the cells were incubated for an additional 24, 48 or 72 h after irradiation. The protective effects of EGCG were measured (**A**) 24, (**B**) 48 or (**C**) 72 h after irradiation. (**D**) The effects of different pretreatment times of 50 µM EGCG on HaCaT cell viability were determined. Data are presented as the mean ± SEM of three independent experiments. An asterisk indicates *P* < 0.05, compared with the DMSO-pretreated control group.

In this study, we first investigated the effect of EGCG on the viability of human skin HaCaT cells by MTT assay. As shown in Fig. 1A, B and C, EGCG did not exhibit a cytotoxic effect at doses up to 75 µM. Compared with DMSO-pretreated cells, pretreatment with 25–75 µM EGCG resulted in a significant increase in cell viability of HaCaT cells 24 h after 20-Gy X-ray irradiation (Fig. 1A). Similar results were observed for the EGCG-pretreated cells 48 and 72 h after irradiation (Fig. 1B and C). Taken together, the pretreatment of EGCG at 50 µM showed consistently increased maintenance of cell viability from 24–72 h after irradiation. Therefore, 50 µM EGCG was selected for further experiments. To select the optimal time of administration of EGCG, we tested the effect with different pretreatment times. As shown in Fig. 1D, pretreatment of EGCG 1 h prior to irradiation showed the most pronounced protective effect and thus this pretreatment time was selected for further experiments. These results all clearly suggest that pretreatment with EGCG significantly increases the viability of HaCaT cells after ionizing radiation.

### EGCG conferred radioresistance on HaCaT cells

**Fig. 2.**
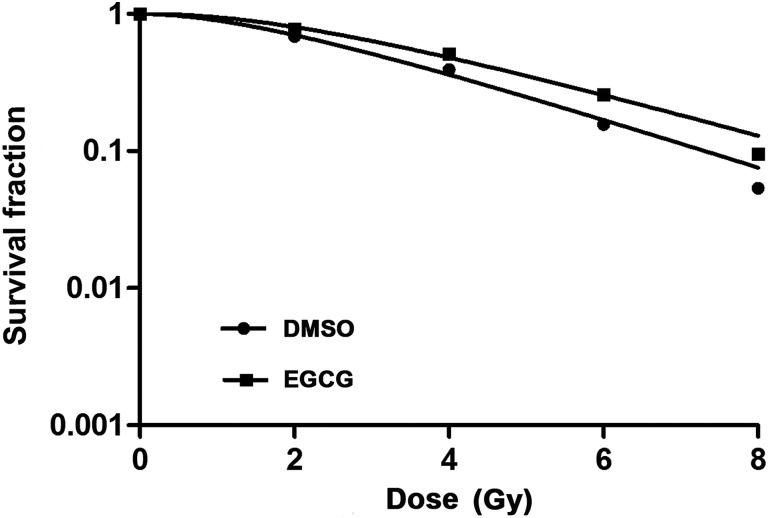
Clonogenic survival of HaCaT cells following radiation. HaCaT cells were seeded in six-well plates, pretreated with DMSO or 50 µM EGCG, and subjected to a range of doses of ionizing radiation.

To investigate the effect of EGCG on the radiosensitivity of HaCaT cells, we performed an *in vitro* clonogenic cell survival assay. HaCaT cells were pretreated with EGCG 1 h before irradiation. The results showed that EGCG-pretreated cells exhibited higher clonogenic survival rates than control cells pretreated with only DMSO (Fig. 2). According to the multitarget model, we obtained the main parameters of the dose–survival curves of HaCaT cells. The mean lethal dose (D_0_) of radiation for the control cells and for the EGCG pretreated group was 2.43 Gy and 2.63 Gy, respectively, while the quasi-threshold dose (Dq) was 1.78 Gy and 2.53 Gy, respectively. HaCaT cells treated with radiation plus 50 µM EGCG exhibited a SER of 0.92, compared with cells treated with radiation plus an equivalent concentration of DMSO. The results also revealed that interaction between EGCG pretreatment and radiation was statistically significant (*P* < 0.05) for HaCaT cells, demonstrating that EGCG confers radioresistance to HaCaT cells.

### EGCG reduced the generation of radiation-induced intracellular ROS

**Fig. 3.**
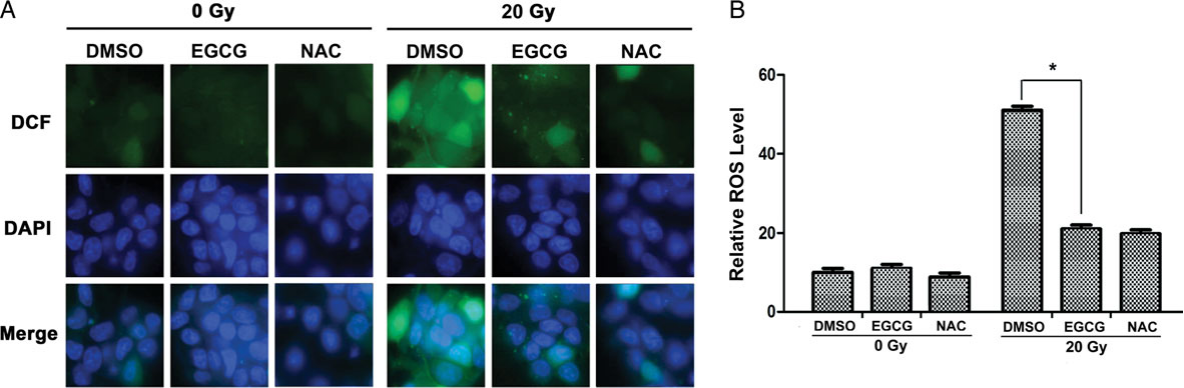
EGCG protects HaCaT cells from radiation-induced ROS production. HaCaT cells were pretreated with 50 µM EGCG for 1 h and then X-ray irradiated or treated as control. After incubation for 24 h, 10 μM DCF-DA probe was added for 30 min, which reacted with cellular ROS and was metabolized into fluorescent DCF. (**A**) Representative images of fluorescent signals by a fluorescence microscope (Leica SP2, Heidelberg, Germany) under equivalent conditions. (**B**) The histogram profile of the relative ROS levels of the four indicated groups. At least 300 cells were counted from 10 randomly chosen ﬁelds of view. The percentages of the fluorescent intensities of the cells were quantified using ImageJ image analysis software (MD, USA). Data are mean ± SEM. An asterisk indicates *P* < 0.05.

EGCG has been shown to confer cytoprotection against ROS-induced oxidative stresses [26]. We therefore investigated whether EGCG could scavenge the radiation-induced intracellular ROS using the DCF-DA assay. As shown in Fig. 3A and B, 24 h after exposure to 20-Gy X-ray irradiation, the fluorescence intensity of DCF in HaCaT cells was significantly increased, while the background ROS levels in HaCaT cells were low without irradiation. However, 50 µM EGCG significantly decreased the radiation-induced generation of ROS, suggesting that EGCG pretreatment could significantly inhibit the steady state levels of ROS induced by X-ray irradiation.

### Reduced apoptosis of HaCaT cells by EGCG after X-ray irradiation

**Fig. 4.**
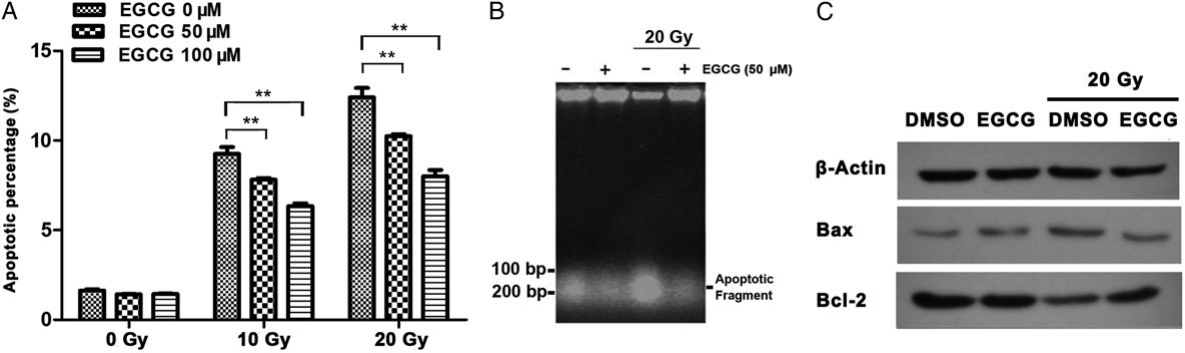
EGCG alleviated radiation-induced apoptosis in HaCaT cells. (**A**) Cells were pretreated with EGCG for 1 h before being treated with 10 or 20 Gy of irradiation. Cells were incubated for another 48 h and then digested by trypsin. The apoptotic cell percentage was detected by flow cytometry. An asterisk indicates *P* < 0.05. (**B**) DNA was extracted from HaCaT cells with or without being pretreated with EGCG before irradiation. Isolated DNA was subjected to 1.2% agarose electrophoresis at 35 V for 4 h. (**C**) Western blot was performed to detect the protein level of the apoptosis-related proteins Bcl-2 and Bax.

Using Annexin V/PI staining, the percentage of apoptotic cells before and after exposure to 20-Gy X-ray irradiation was detected using flow cytometry. The apoptotic cell percentage was not significantly altered in the EGCG-pretreated cells compared with the DMSO-pretreated cells without irradiation (Fig. 4A). Additionally, we detected DNA fragmentation to confirm apoptotic cell death induced by radiation. The results showed that radiation exposure clearly increased DNA fragmentation (Fig. 4B), indicating increased numbers of cells undergoing apoptosis. However, EGCG pretreatment of cells reduced the apoptosis percentage and the DNA fragments, compared with the control cells after irradiation. The apoptosis percentage was reduced by EGCG under non-irradiated conditions. Moreover, EGCG pretreatment upregulated the anti-apoptotic protein Bcl-2 and downregulated the pro-apoptotic protein Bax.

### EGCG pretreatment maintained mitochondrial mass

**Fig. 5.**
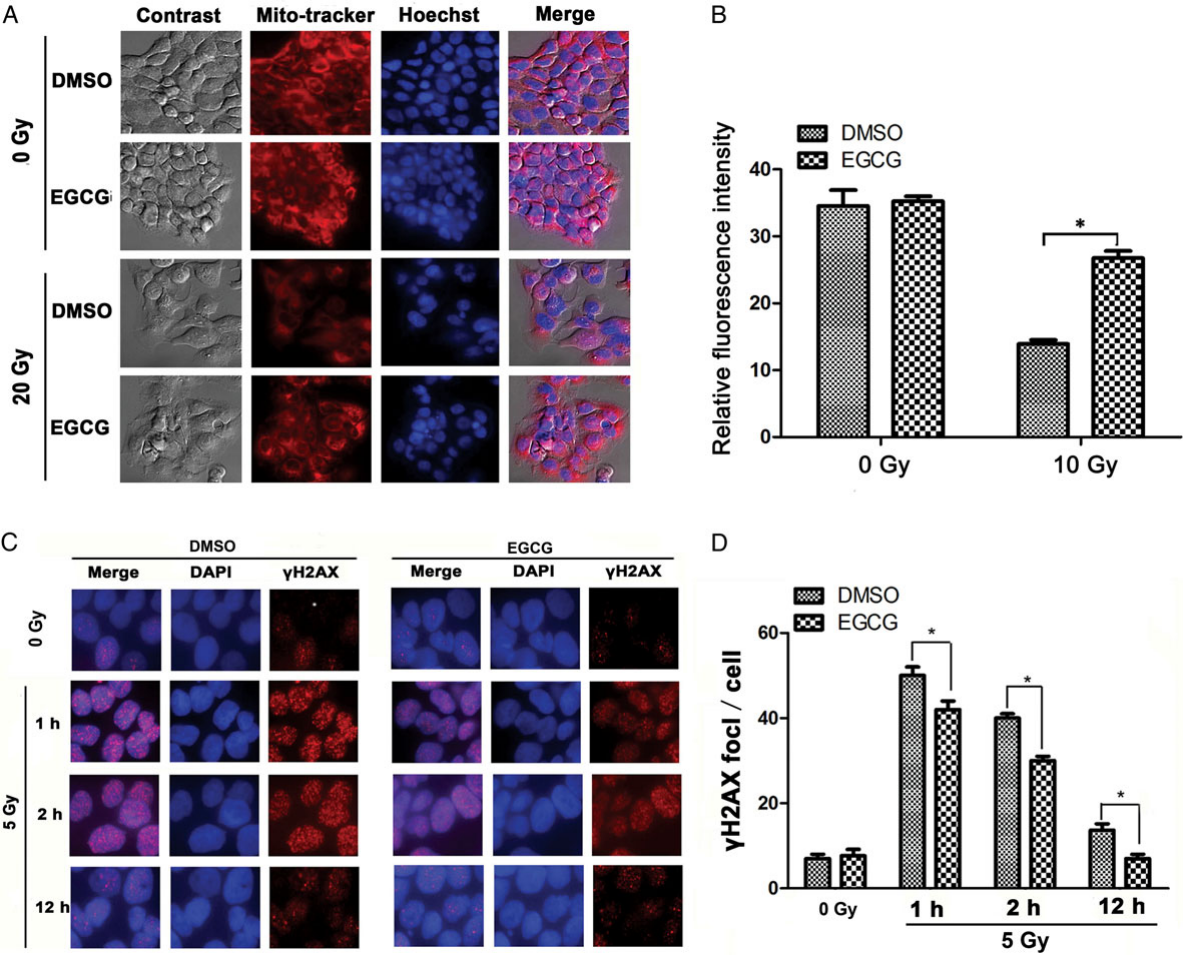
Effect of EGCG on mitochondrial mass and γH2AX foci in HaCaT cells. (**A**) HaCaT cells were X-ray irradiated after EGCG pretreatment for 1 h. Mito-Tracker Red was used to detect the mitochondrial membrane integrity. The nuclei of the cells were stained with Hoechst for another 30 min and analyzed by fluorescence microscopy ( × 600 magnification). (**B**) The relative fluorescence intensity of mitochondria in HaCaT cells was calculated with ImageJ image analysis software (Bethesda, MD, USA). At least 300 cells were counted from 10 randomly chosen ﬁelds of view. Data are mean ± SEM. An asterisk indicates *P* < 0.05. (**C**) Immunofluorescence of γH2AX foci (red) in untreated HaCaT cells and in cells irradiated with 5-Gy X-ray irradiation for an additional 1, 2 or 12 h at 37°C. Nuclei were stained with DAPI (blue). Images were acquired using a fluorescent microscope (Leica SP2, Heidelberg, Germany). (**D**) The average number of foci per cell was obtained from three independent experiments. At least 300 cells were counted from 10 randomly chosen ﬁelds of view. Data are mean ± SEM. An asterisk indicates *P* < 0.05.

Radiation-induced ROS may also attack macromolecules such as mitochondrial DNA and membrane proteins in mitochondria, resulting in the impairment of mitochondrial function [27]. Damaged mitochondrial DNA and other components are likely to affect mitochondrial division and cellular homeostasis [28]. To explore whether EGCG protected the mitochondria of HaCaT cells, Mito-tracker Red probe was used to assess the mass of the mitochondria. As shown in Fig. 5A and B, weak red fluorescence staining was observed following ionizing radiation, indicating destruction of mitochondrial mass of HaCaT cells. Pretreatment with EGCG restored the red fluorescence, indicating its maintenance of the mitochondrial mass.

### EGCG decreased the number of γH2AX foci

We further explored whether EGCG influences the dynamic repair process of DNA double-strand breaks (DSBs) induced by radiation. DSBs are considered to be the most significant lesion caused by ionizing radiation. DNA damage was measured by immunostaining of phosphorylated H2AX protein (γ-H2AX), which is recruited to nuclear structures termed foci. γ-H2AX foci occurred immediately after irradiation, reached a high point at 1 h after 5-Gy irradiation and remained present for nearly 24 h. We measured the γ-H2AX foci 1, 2 and 12 h post-irradiation. Compared with the control cells, EGCG pretreatment of HaCaT cells significantly reduced the γ-H2AX foci number (Fig. 5C and D), indicating that cells pretreated with EGCG had significantly decreased DNA damage.

### EGCG activates HO-1 in HaCaT cells via transcriptional activation

**Fig. 6.**
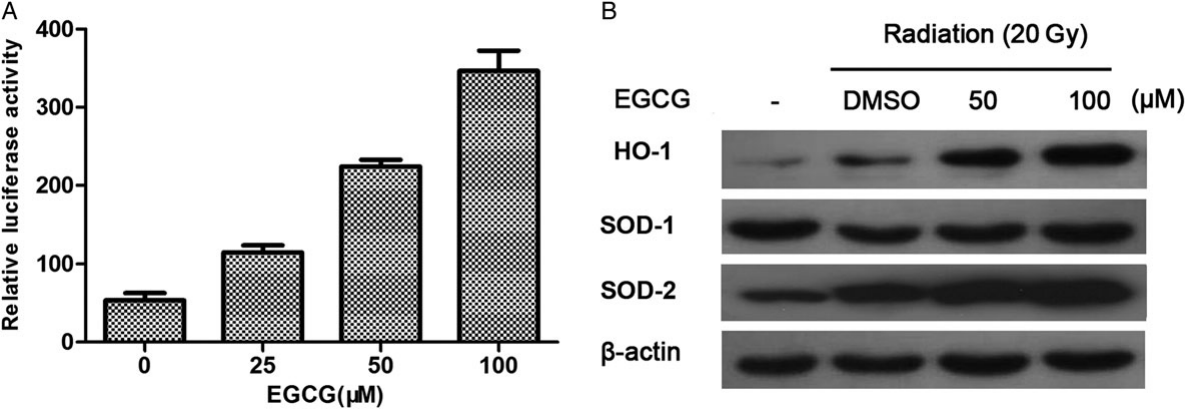
EGCG upregulated HO-1 via transcriptional activation. (**A**) A luciferase reporter containing the *HO-1* promoter was transfected into HaCaT cells, which were then exposed to the indicated concentration of EGCG for an additional 24 h. Data are mean ± SEM. (**B**) The effect of EGCG treatment plus irradiation on the protein expression of HO-1, SOD1 and SOD2. HaCaT cells were X-ray irradiated after EGCG pretreatment for 1 h. Western blot was performed to detect the protein level of HO-1, SOD1 and SOD2.

To investigate whether EGCG modulates transcriptional regulation of *HO-1*, its promoter fragment was cloned into the pGL3 vector upstream of a luciferase reporter gene and assessed for transcriptional activation in HaCaT cells. As shown in Fig. 6A, EGCG treatment was found to enhance the reporter activity in a dose-dependent manner, indicating transcriptional activation. Western blot analysis confirmed that EGCG treatment induced HO-1 overexpression in a dose-dependent manner (Fig. 6B). Furthermore, we found that EGCG also induced the expression of mitochondrial-localized SOD2 but not SOD1.

### HO-1 inhibition reversed the protective effect of EGCG

**Fig. 7.**
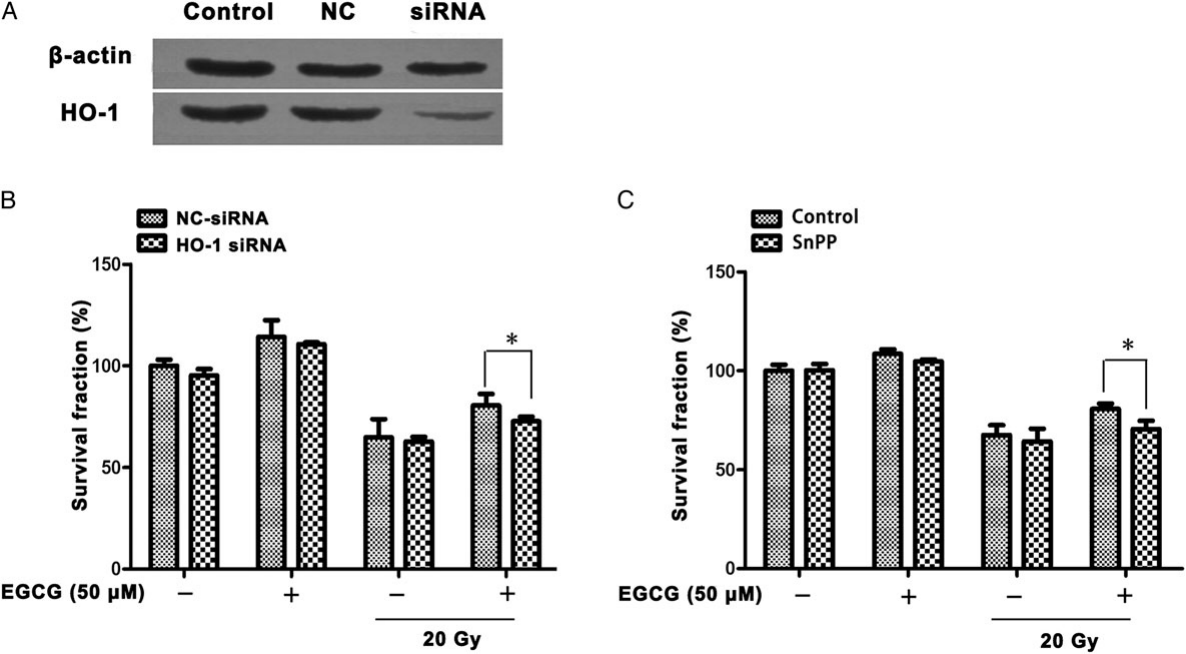
Inhibition of HO-1 by SnPPIX or siRNA decreased radioprotection of EGCG in HaCaT cells. (**A**) Transfection of HO-1 targeting siRNA downregulated HO-1 protein. HO-1 expression was determined by Western blot analysis 48 h after transfection with HO-1 siRNA. (**B**) The effect of HO-1 silencing on the viability of HaCaT cells with or without irradiation. Cells were transfected with the indicated siRNA and then pretreated with EGCG (50 µM) before 20 Gy of irradiation. (**C**) The effect of HO-1 inhibition on the viability of HaCaT cells with or without irradiation. Cells were incubated with SnPPIX (10 µM) and EGCG (50 µM) for 1 h, then X-ray irradiated. Cell viability was determined by MTT assay. Data are presented as mean ± SEM. An asterisk indicates *P* < 0.05.

To demonstrate that the protective role of EGCG was mediated by HO-1, we knocked down HO-1 protein expression via transfection with an siRNA targeting HO-1 (Fig. 7A). As shown in Fig. 7B, knockdown of HO-1 increased the sensitivity of HaCaT cells to radiation. To confirm this result, we treated cells with SnPPIX, which is a specific inhibitor of HO-1. After the addition of SnPPIX, 50 µM EGCG pretreatment showed a slight radioprotective effect against irradiation (Fig. 7B), suggesting a pivotal role of HO-1 in the protective effect of EGCG.

## DISCUSSION

Skin injury caused by ionizing radiation is one of the most common complications of radiation oncology treatment [29]. Chronic radiation-induced skin damage is related to ROS generated in skin that is exposed for an extended time and apoptosis that is promoted by inflammatory reaction factors. In this study, we showed that EGCG enhanced the values of D_0_ and Dq in HaCaT cells after irradiation. These results indicate that EGCG decreased the radiosensitivity of HaCaT cells and enhanced their capacity for sublethal damage repair. We also found that pretreatment with 50 µM EGCG significantly enhanced cell viability, maintained cell mitochondrial mass and decreased ROS and apoptosis induced by X-ray irradiation of skin HaCaT cells. However, the reduction in apoptotic rate was greater with 100 μM EGCG than with 50 μM EGCG, which is inconsistent with the results from the MTT assay that indicated 50 μM EGCG showed the best protective effect. This discrepancy may be due to different evaluation indices for the MTT assay and the apoptosis analysis. Considering the proliferation promoting effect of EGCG to non-irradiated normal tissues, a dose of 50 μM EGCG would be more applicable.

Numerous studies have indicated that radiation-induced cytotoxicity is triggered by ROS, which regulate multiple cellular processes [30]. As a consequence, biological macromolecules such as DNA, RNA and proteins are destroyed. Moreover, loss of enzyme activity, cell cycle disorder, cell apoptosis and cell death can also occur [31, 32]. Thus the imbalance between ROS generation and elimination contributes to disease initiation and development [33]. Most intracellular ROS are generated via mitochondrial electron transport [34]. EGCG is the major polyphenolic constituent in green tea and is also a powerful antioxidant and free radical scavenger with the ability to modulate cell growth, cell cycle and apoptosis in human cells [35, 36]. In the present study, we measured the ROS levels by DCF-DA and found that EGCG pretreatment could suppress radiation-induced ROS and affect oxidative status. Moreover, EGCG was found to preferentially induce apoptosis in tumor cells but not normal cells [37], which intensified its application for cancer patients especially for those receiving radiotherapy. *In vitro* and *in vivo* experiments have shown that EGCG can reduce UV-induced skin damage [38]. The present work illustrates a radioprotective role for EGCG, which expands the known beneficial effects of EGCG.

A variety of factors (such as hypoxia, genetic damage, extreme oxidation pressure, and radiation) trigger the initiation of the mitochondrial apoptotic pathway, leading to an increase in mitochondrial permeability. Thus, maintaining mitochondrial mass and function is important for preventing the progression of many diseases [39]. EGCG has been shown to prevent mitochondrial damage induced by isoproterenol [40]. We showed that pretreatment of EGCG maintained mitochondrial mass and reduced apoptosis after irradiation, which may be attributed to the induction of SOD2. SOD2, also known as MnSOD, is exclusively located in mitochondria and plays a critical role in protection against ionizing radiation in mammalian cells [41]. The mitochondria-dependent apoptotic pathway is regulated by proteins such as the pro-apoptotic Bax and anti-apoptotic Bcl-2 proteins [42]. Our results show that EGCG pretreatment suppressed apoptosis and enhanced the Bcl-2/Bax ratio, which is consistent with its protective role for mitochondria.

The application of EGCG has been reported to activate multiple pathways, including the PI3K-Akt, MEK/ERK and FOXO pathways [43, 44]. Induction of the cytoprotective protein HO-1 by EGCG has also been well established in multiple carcinoma cells [45] and endothelial cells [46]. HO-1 is a ubiquitous, redox-sensitive and inducible stress protein that catalyzes heme to produce carbon monoxide and bilirubin, representing an adaptive response to oxidative damage [47, 48]. Several mechanisms such as anti-oxidant function and maintenance of microcirculation, anti-apoptotic and anti-inflammatory properties may contribute to the cytoprotection of HO-1 overexpression [48–50]. We have previously reported that overexpression of antioxidant heme HO-1 ameliorates radiation-induced skin injury in rats [51]. In this study, we confirmed that EGCG treatment boosts HO-1 expression in human skin HaCaT cells in a dose-dependent manner via transcriptional activation. Thus, we postulate that the radioprotective effect of EGCG on HaCaT cells may in part be attributed to HO-1 overexpression. To validate this hypothesis, we inhibited HO-1 both via transfection of an siRNA targeting HO-1 and via treatment with a competitive inhibitor of HO-1 (SnPPIX) prior to treatment with EGCG. We found that the protective effect of EGCG was attenuated, indicating that HO-1 mediated the protective function of EGCG in response to X-ray irradiation.

## CONCLUSION

In this study, we found that in skin HaCaT cells irradiated by X-rays, the application of EGCG significantly enhanced viability, maintained cell mitochondrial mass and decreased apoptosis induction. ROS and γH2AX foci in HaCaT cells were significantly reduced when pretreated with EGCG prior to irradiation. Moreover, HO-1 knockdown or the HO-1 specific inhibitor tin protoporphyrin (SnPPIX) reversed the protective role of EGCG, indicating the pivotal role of HO-1. Taken together, these results suggest that the application of EGCG offers a new strategy for protecting skin against ionizing radiation.

## FUNDING

Funding to pay the Open Access publication charges for this article was provided by the National Natural Science Foundation of China (81102078).
